# Work engagement status of registered nurses in pediatric units in Saudi Arabia: A cross‐sectional study

**DOI:** 10.1371/journal.pone.0283213

**Published:** 2023-03-17

**Authors:** Manal F. Alharbi, Reham Z. Alrwaitey

**Affiliations:** 1 Maternal & Child Health Nursing Department, College of Nursing, King Saud University, Riyadh, Saudi Arabia; 2 Nursing Education Administration, King Salman Bin Abdulaziz Medical City, Ministry of Health, Madinah, Saudi Arabia; Birla Institute of Technology and Science, Pilani, INDIA

## Abstract

**Background:**

This study aimed to evaluate the work engagement status of registered pediatric nurses and its relationship with personal- and work-related variables in selected hospitals. Personal- and work-related factors generally influence work engagement. However, data on work engagement in pediatric clinical practice are limited.

**Methods:**

This study employed a cross-sectional design, including 230 registered nurses working in pediatric units in Madinah, Saudi Arabia. A non-probability approach (convenience sampling) was adopted in recruiting the sample. Eight personal- and work-related variables were examined using the Utrecht Work Engagement Scale shortened 9-item version.

**Results:**

The overall mean scale score was 4.54 (standard deviation = 0.95). The dedication subscale showed the highest mean score (4.84), followed by the absorption (4.48) and vigor subscales (4.29). A higher work engagement score was associated with an older age (H = 17.892; p < 0.001), a non-Saudi nationality (Z = 5.724; p < 0.001), a higher educational level (Z = 3.178; p = 0.001), and a long duration of experience (>10 years) (H = 18.435; p < 0.001). No significant differences were observed between the total scale score according to marital status (p = 0.077), current working unit (p = 0.063), and current working hours (p = 0.067).

**Conclusions:**

Among registered pediatric nurses, work engagement is relatively high but is average in terms of the vigor component. To our knowledge, this research is the first to explore how work environment affects work engagement among pediatric nurses in Madinah, Saudi Arabia.

## Introduction

Nursing is the profession that comes in direct coordination with patients [1]. Pediatric nurses have a strong impact on children’s clinical experiences. Pediatric nursing care is challenging, as the care provided to different age groups (from infants to adolescents) requires professional management during distinctive growth-related stages of motor, physical, emotional, and cognitive developments. Such nursing care is based on family-centered care philosophy [2]. Accordingly, pediatric nurses should be skilled in managing patients and adopt a supportive attitude toward their families [3]. In pediatric nursing, attitudes toward patient engagement has a significant impact on nurses well-being [4] as well as the quality of care they provide [5]. A significant number of pediatric nurses have been reported to have moderate-to-high levels of emotional exhaustion and depersonalization along with low levels of personal accomplishment [6]. Within pediatric units, nurses may face more challenges. Children are seen as the most vulnerable patient group and require empathetic engagement to tackle complexities in the relationships with their families [7,8]. Pediatric nurses experience high levels of stress at their work environment owing to the complicated care needs of children and family stress [8,9]. Although nurses in pediatric units may gain satisfaction from providing compassionate care to pediatric patients and their families, they are also at risk of experiencing fatigue, affecting their performance and work engagement [10].

### Work engagement

The concept of work engagement is based on the capability of employees to express their cognitive, physical, and emotional well-being into work during task performance [11]. Work engagement has recently emerged as an antipode of burnout [12]. It is a positive state of mind characterized by three scopes: dedication, vigor, and absorption [12]. Resilience and high energy levels related to work and willingness to invest effort and learn in difficult scenarios are described under vigor [12]. A sense of motivation, pride, and inspiration to handle challenge is referred to as dedication [12]. Full engrossment in an assigned task or responsibility comes under absorption [13]. Among healthcare providers, work engagement refers to the involvement of professional skills, interest, energy, and physical capabilities exhibited in fulfillment of their responsibilities [14]. A systematic review examined the antecedents and outcomes of work engagement in professional nursing practice and identified 17 outcomes of work engagement categorized into performance and care outcomes, professional outcomes, and personal outcomes [15]. More recent reviews have also shown that work engagement influences the quality of care and patient safety [16,17]. These results corroborate the importance of enhancing work engagement to improve the performance of nurses, which has been the focus of many studies.

Work engagement arises from both human and environmental sources. It generates positive outcomes and is regarded as a predictor of job satisfaction along with social support. A study that investigated 1,024 nurses confirmed the association between work engagement and ability [18]. Work engagement derives from psychological empowerment, which is a personal response to managerial interventions that create empowering work environments [19]. The literature on burnout describes employee engagement as a positive antipode of burnout characterized by involvement, energy, and effectiveness [20].

### Key predictors of nurse work engagement

Nurses’ professional and organizational performances are greatly related to the quality of care. Research has shown several organization-, task-, and personal-related factors affecting nurses’ involvement in assigned responsibilities. One of these factors include age: A study conducted in China reported a significant association of age with work engagement, with 3% variance in work commitment [21]. Another study performed in Saudi Arabia (SA) among 980 nurses reported significant but weak positive correlations between nurses’ age, education, and total work engagement scores [22]. Nevertheless, other previous literature has described inconsistent relationships between age and work engagement, requiring further investigations on this issue [23]. Some challenging factors include staff burnout and low turnover, which reduce the engagement of nurse employees in pediatric care units owing to secondary traumatic stress or compassion fatigue. A job redesign concept through job crafting resulted in 57% work engagement variance in SA [24]. A study conducted in Oman and United Arab Emirates among nurses reported that 88.3% of nursing professionals were aged between 30 and 49 years and found a weak correlation between work engagement and demographic details of healthcare providers [25]. Considering years of nursing experience, a study found a significant difference in gender and support, opportunity, and organizational growth and relationship, indicating higher empowerment levels among male nursing faculty. However, the effect of nationality, age, and educational qualification was found to be insignificant [26]. Another study in SA among nurses reported a significant negative correlation between turnover satisfaction and age and a positive correlation between nationality and dedication toward work [27].

Another challenge is active nursing involvement in pediatric clinical intervention programs and special education provision for children. This could be overcome through introduction of an evidence-based teaching practice in healthcare organizations. An introduction of staff resilience programs in nursing education plans has been found to improve compassion satisfaction and psychological well-being of care providers in pediatric intensive care units [28]. A study in Poland demonstrated an association between nursing education and work satisfaction and a particularly significant association between personal satisfaction and job satisfaction among nursing managers [29]. A study in Indonesia reported a significant and positive correlation between continuing professional development through a nursing career ladder system and satisfaction in work engagement [30].

Considering the above-indicated literature, it is imperative to examine pediatric nurses’ work engagement to provide comprehensive, ethical, and safe care to children. Yet, most of the research conducted on work engagement does not separately evaluate nurses working in pediatric departments. The retention and engagement of professional registered nurses in pediatric departments are a great challenge among healthcare organizations in SA in improving the quality of care and fully mobilizing productive resources. Owing to scarce data in SA, it is essential to examine the association between the predictors of work engagement among pediatric nurses. To reduce the turnover percentage of nurses, researchers must conduct studies in pediatric departments to evaluate the work engagement and predictors that influence nurses’ work-related positive state of mind while providing dedicated care to young populations. Therefore, this study aimed to examine the level of work engagement among registered pediatric nurses and its relationship with personal- and work-related characteristics in selected hospitals.

### Hypothesis

H^0^: There is no significant difference between work engagement (dedication, absorption, and vigor) and personal- and work-related characteristics among nurses in pediatric units.

H^1^: There is a significant difference between work engagement (dedication, absorption, and vigor) and personal- and work-related characteristics among nurses in pediatric units.

## Materials and methods

### Study design and participants

This quantitative cross-sectional descriptive study was conducted at the pediatric department of two government hospitals in Madinah City, SA, from January to April 2021. The study sample size was calculated using a specific formula for a considerable population [31]. The assumed values included a confidence level of 95%, Z-score of 1.96, margin error of 0.05%, and standard deviation (SD) of 50% as a measure of variation. Accordingly, the sample size needed was 384 pediatric nurses. Participants were recruited prospectively using convenience sampling. Of the 384 initial participants, 230 responded, yielding a response rate of 59.8%; these participants were then recruited in the study. The target study population was nurses employed at two government hospitals in Madinah City with any nationality, currently working directly as bedside nurses for pediatric patients. All nurses working in other departments were excluded from the study. After explaining the study objective to the participants, we collected informed consent through a survey tool in an electronic form with a statement indicating form submission and provision of informed consent by clicking the “Continue” button on the first page of the survey. Through the survey tool, the participants were provided with the research objective, purpose, and instructions. The details of all study participants were kept strictly confidential. The study was initiated after approval from ethics committee (Approval No: KSU- E-20-4945) and conducted as per 1964 Declaration of Helsinki guidelines.

### Study questionnaire

A self-administered electronic questionnaire with three sections was prepared. The questionnaire evaluated 17 variables, including 4 socio-demographic, 4 work profile, and 9 work engagement variables. Section I included the demographic variables (sex, age, marital status, and nationality); section II, work profile variables (educational level, work experience, name of the unit, and working hours); and section III, work engagement variables according to the Utrecht Work Engagement Scale shortened 9-item version (UWES-9S) developed by Schaufeli et al. [32]. The UWES-9S is a nine-item scale with three dimensions: vigor, dedication, and absorption. The scores are classified as follows: very low, low, average, high, and very high. The responses to all dimensions are scored on a 7-point frequency rating scale ranging from 0 (*never*) to 6 (*always*) [15]. The mean score for the three UWES-9S subscales is computed by adding the scores on the particular scale and dividing the sum by the number of items of the subscale involved [15]. The UWES-9S is a valid and reliable tool for assessing work engagement; it has a Cronbach α of 0.885 [15]. In the current study, the work engagement scale was reliable (α = 0.93) and particularly excellent for the vigor (α = 0.91), dedication (α = 0.94), and absorption dimensions (α = 0.92). The UWES-9S items were piloted in a small group of participants to verify their clarity; among this group, 15 participants expressed no difficulties in understanding the items or answering the scale. The participants in the pilot study were excluded from the main study.

### Statistical analysis

All categorical variables were presented as numbers and percentages and all continuous variables as means and SDs. The overall UWES-9S score was compared with the socio-demographic characteristics of the nurses using the Mann–Whitney Z-test and Kruskal–Wallis H-test. A post hoc analysis was conducted to determine the multiple mean differences in the overall UWES-9S score in relation to age and work experience. Pearson correlation coefficient was used to determine the correlation between the total UWES-9S and subscale scores. Normality of data was evaluated using the Shapiro–Wilk test as well as the Kolmogorov–Smirnov test. The UWES-9S score was found to have a non-normal distribution; thus, non-parametric tests were applied. A p-value of <0.05 was considered statistically significant. The data were analyzed using the Statistical Package for the Social Sciences version 26 (IBM Corp., Armonk, NY, USA).

## Results

### Participant characteristics

A total of 230 female pediatric nurses completed the survey. As described in Table 1, half of the nurses were aged between 31 and 40 years (50.9%) and were single (50.9%). The majority were non-Saudi nurses (70%), and nearly all had bachelor’s degrees (81.3%). Nearly 70% were working in a specialty area, with approximately 56.1% working up to 12 hours per day. In addition, 36.5% had 10–15 years of working experience.

**Table 1:** Socio-demographic characteristics of the female pediatric nurses (n = 230).

Characteristics	n (%)
Age	
• 20–30 years	76 (33.0%)
• 31–40 years	117 (50.9%)
• >40 years	37 (16.1%)
Marital status	
• Single	117 (50.9%)
• Married	113 (49.1%)
Nationality	
• Saudi	69 (30.0%)
• Non-Saudi	161 (70.0%)
Educational level	
• Diploma	43 (18.7%)
• Bachelor	187 (81.3%)
Employment specialty	
• Specialty unit	160 (69.6%)
• General unit	70 (30.4%)
Current working hours	
• 8-hour shift	101 (43.9%)
• 12-hour shift	129 (56.1%)
Work experience	
• 1–5 years	75 (32.6%)
• 5–10 years	71 (30.9%)
• 10–15 years	84 (36.5%)

### UWES-9S score

The descriptive statistics of the total UWES-9S and subscale scores are shown in Table 2. The mean overall UWES-9S score was 4.54 (SD = 0.95). Among the UWES-9S subscales, the dedication subscale showed the highest mean score (4.84), followed by the absorption (4.48) and vigor subscales (4.29). In terms of the UWES-9S level, most nurses were found to have average overall UWES-9S (50%), vigor (42.6%), dedication (54.8%), and absorption levels (57.4%).

**Table 2:** Descriptive statistics of the UWES-9S scores (n = 230).

UWES-9S subscale	n (%)
**Vigor subscale score (mean ± SD)**	**4.29 ± 1.00**
• Very low (<5^th^ percentile)	8 (3.5%)
• Low (≥5^th^ percentile)	32 (13.9%)
• Average (≥25^th^ percentile)	98 (42.6%)
• High (≥75^th^ percentile)	71 (30.9%)
• Very high (≥95^th^ percentile)	21 (9.1%)
**Dedication subscale score (mean ± SD)**	**4.84 ± 1.03**
• Very low (<5^th^ percentile)	8 (3.5%)
• Low (≥5^th^ percentile)	19 (8.3%)
• Average (≥25^th^ percentile)	126 (54.8%)
• High (≥75^th^ percentile)	25 (10.9%)
• Very high (≥95^th^ percentile)	52 (22.6%)
**Absorption subscale score (mean ± SD)**	**4.48 ± 1.05**
• Very low (<5^th^ percentile)	13 (5.7%)
• Low (≥5^th^ percentile)	40 (17.4%)
• Average (≥25^th^ percentile)	132 (57.4%)
• High (≥75^th^ percentile)	19 (8.3%)
• Very high (≥95^th^ percentile)	26 (11.3%)
**UWES score (mean ± SD)**	**4.54 ± 0.95**
• Very low (<5^th^ percentile)	9 (3.9%)
• Low (≥5^th^ percentile)	43 (18.7%)
• Average (≥25^th^ percentile)	115 (50.0%)
• High (≥75^th^ percentile)	52 (4.8%)
• Very high (≥95^th^ percentile)	11 (4.8%)

UWES-9S, Utrecht Work Engagement Scale shortened 9-item version; SD, standard deviation.

**Fig 1:**
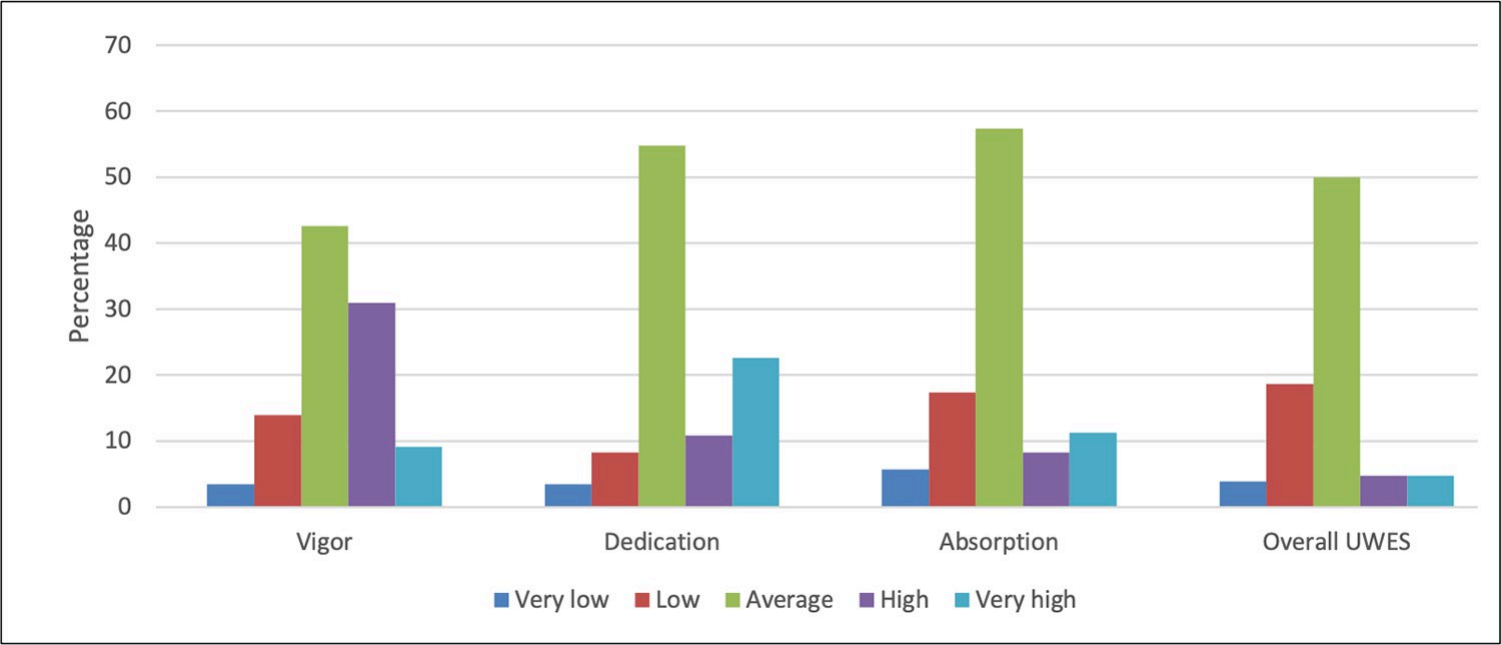
Level of work engagement and its subscales.

For the establishment of statistical norms for the UWES-9S, the five categories—very low, low, average, high, and very high—were used in accordance with the test manual of the Utrecht Work Engagement Scale [33]. Level of work engagement and its subscales are shown in (Fig 1).

As shown in Table 3, the Pearson correlation coefficient indicated a positive and highly significant correlation between the total UWES-9S and subscale scores (p < 0.001). This demonstrated that an increase in the total UWES-9S score was correlated with an increase in the subscale scores, which was also reflected in each pairwise comparison.

**Table 3:** Correlation between the total UWES-9S and subscale scores (n = 230).

UWES-9S subscale	Vigor	Dedication	Absorption	Total UWES-9S
Vigor	1			
Dedication	**0.810** ^**^	1		
Absorption	**0.754** ^**^	**0.807** ^**^	1	
Total UWES	**0.919** ^**^	**0.941** ^**^	**0.923** ^**^	1

^**^Significant at p = 0.01 (two-tailed).

UWES-9S, Utrecht Work Engagement Scale shortened 9-item version.

### Relationship between the UWES-9S score and personal- and work-related profiles

In the analysis of the differences in the total UWES-9S score in relation to the socio-demographic characteristics of the nurses (Table 4), a higher UWES-9S score was found to be associated with an older age (H = 17.892; p < 0.001), a non-Saudi nationality (Z = 5.724; p < 0.001), a higher educational level (Z = 3.178; p = 0.001), and a long duration of experience (>10 years) (H = 18.435; p < 0.001). No significant differences were observed between the total UWES-9S score according to marital status (p = 0.077), current working unit (p = 0.063), and current working hours (p = 0.067).

**Table 4:** Differences in the UWES-9S score and socio-demographic characteristics of the female pediatric nurses (n = 230).

Characteristics	UWES-9S score (6) Mean ± SD	Z/H-value	p-value
Age^a^			
• 20–30 years	4.25 ± 0.94	H = 17.892	**<0.001** ^**^
• 31–40 years	4.56 ± 0.89		
• >40 years	5.05 ± 0.99		
Marital status^b^			
• Single	4.43 ± 0.91	Z = 1.768	0.077
• Married	4.65 ± 0.99		
Nationality^b^			
• Saudi	4.00 ± 0.91	Z = 5.724	**<0.001** ^**^
• Non-Saudi	4.77 ± 0.88		
Educational level^b^			
• Diploma	4.13 ± 0.97	Z = 3.178	**0.001** ^**^
• Bachelor	4.63 ± 0.93		
Employment specialty^b^			
• Specialty unit	4.59 ± 0.96	Z = 1.862	0.063
• General unit	4.39 ± 0.93		
Current working hours^b^			
• 8-hour shift	4.41 ± 0.98	Z = 1.832	0.067
• 12-hour shift	4.64 ± 0.93		
Work experience^a^			
• 1–5 years	4.32 ± 0.87	H = 18.435	**<0.001** ^**^
• 5–10 years	4.34 ± 1.01		
• 10–15 years	4.89 ± 0.88		

^a^P-value calculated using the Kruskal–Wallis H-test.

^b^P-value calculated using the Mann–Whitney Z-test.

**Significant at p < 0.05.

UWES-9S, Utrecht Work Engagement Scale shortened 9-item version; SD, standard deviation.

In the post hoc analysis (Table 5), there was a significant difference observed in the total UWES-9S score between the participants aged 20–30 years and >40 years (p < 0.001) and between those aged 31–40 years and >40 years (p = 0.018). Similarly, we found a significant difference in the total UWES-9S score between those with 1–5 years and >10 years of experience (p < 0.001) and between those with 5–10 years and >10 years of experience (p = 0.001).

**Table 5:** Post hoc analysis of the multiple differences in the UWES-9S score in relation to age and work experience (n = 230).

**(I) Age**	**(J) Age**	**Mean difference (I−J)**	**Standard error**	**p-value**	**95% confidence interval**	
					**Lower bound**	**Upper bound**
20–30 years	31–40 years	−0.31074	0.13579	0.069	−0.6382	0.0168
	>40 years	−0.79358*	0.18477	0.000	−1.2392	−0.3480
31–40 years	20–30 years	0.31074	0.13579	0.069	−0.0168	0.6382
	>40 years	−0.48284*	0.17384	0.018	−0.9021	−0.0636
>40 years	20–30 years	0.79358*	0.18477	0.000	0.3480	1.2392
	31–40 years	0.48284*	0.17384	0.018	0.0636	0.9021
**(I) Work experience**	**(J) Work experience**	**Mean difference (I−J)**	**Standard error**	**p-value**	**95% confidence interval**	
					**Lower bound**	**Upper bound**
1–5 years	5–10 years	−0.01515	0.15220	1.000	−0.3822	0.3519
	10–15 years	−0.56974*	0.14603	0.000	−0.9219	−0.2175
5–10 years	1–5 years	0.01515	0.15220	1.000	−0.3519	0.3822
	10–15 years	−0.55459*	0.14818	0.001	−0.9120	−0.1972
10–15 years	1–5 years	0.56974*	0.14603	0.000	0.2175	0.9219
	5–10 years	0.55459*	0.14818	0.001	0.1972	0.9120

Post hoc test conducted using the Dunn–Bonferroni test.

*Significant at p = 0.05.

## Discussion

This study aimed to examine the level of work engagement among registered pediatric nurses and its relationship with personal- and work-related variables in selected hospitals. Using data from hospitals in SA, this research was an effort to fill the knowledge gap and provide baseline data on work engagement of pediatric nurses in association with personal- and work-related variables. To our knowledge, no previous studies have evaluated how these factors interact among pediatric nurses in SA. Herein, the overall UWES-9S score was 4.54 (SD = 0.95), which was considered to be at an average level. A study in China reported an average level of work engagement among nurses, which may be associated with the acute shortage of nurses owing to workload [19,34]. Comparable results were obtained in Spain [35] and Belgium [36]. In the present study, dedication was the top aspect of work engagement, followed by absorption and vigor. The dedication subscale yielded the highest mean score (4.84), while the vigor subscale yielded the lowest mean score (4.29). High-dedication and low-vigor employees have been reported to be more likely to leave their jobs, particularly in the presence of a high workload [35].

The current study found that a higher UWES-9S score was associated with an older age, a non-Saudi nationality, a higher educational level, and a long duration of experience (>10 years). This finding broadly supports previous reports linking age and education with total work engagement scores [22,34,37] and nationality with the dedication dimension of work engagement [27].

Younger nurses have been shown to have low work engagement levels, particularly high vigor and low dedication levels [38], although age has been found to be a positive predictor in another research [22]. However, the effect of nationality, age, and educational qualification was found to be insignificant in another study [26].

The career development theory states that workers aged under 25 years are in the exploratory phase of their career development and need more experience to move forward to gain maturity, which increases with age [39]. Adults aged over 44 years are in the stable phase of employment and have less desire to leave their present employer; as a result, these individuals are more likely to adopt an active attitude and engage in good behavior at their current workplaces [40]. Accordingly, employees aged <25 and >44 years could show increased work involvement in comparison with those aged 25–44 years. Wan et al. found a non-linear link between nurses’ age and level of job engagement, which calls for more research, particularly among diverse populations [21]. However, the effect of nationality, age, and educational qualification was found to be insignificant in another study [26]. There are several possible explanations for this result. The association with an older age and a longer duration of experience (>10 years) may be explained by the fact that older nurses have more work experience and more stable family relationships, leading to higher work engagement levels [34]. Further, a higher educational level helps nurses receive higher salaries and more opportunities at work. In contrast, the association found with a non-Saudi nationality is difficult to explain; however, it might be related to the fact that most Saudi nurses are fresh graduates with a short duration of work experience and are under pressure to establish a family and career, possibly resulting in lower work engagement levels. With the small sample size, these results should be interpreted with caution. The null hypothesis is rejected, and a relationship between work- and personal-related variables significantly affects nurses’ engagement in professional work.

## Limitations

This research has a few limitations that need to be addressed. We recruited only nurses from Madinah City, SA, which resulted in a reasonably small convenience sample that could not represent all pediatric nurses. An additional uncontrolled factor is the heterogeneity of the sample owing to high dependence on foreign nurses and the resulting small number of local trained nurses. Thus, a more representative and expanded sample is needed to confirm our results. In subsequent research, it is important to recruit individuals from a different organizational level to study the possibility of differences. Since this study utilized a cross-sectional design, longitudinal studies must be conducted to validate the results and identify the causality underlying the correlations.

## Clinical implications

The study results may aid healthcare institutions in developing organizational working environments specifically related to pediatric care through selection, training, and retention of professional pediatric nurses, assuring their quality of life and job satisfaction to improve overall patient-centered care systems. This study provides baseline data on pediatric nurses’ interest and involvement in work and the influence of personal- and work-related variables on their work engagement. These data may help national health authorities to prioritize guidelines incorporating evidence-based nursing training programs and involve experienced and young nurses in these educational activities to boost their work engagement. Pediatric patients require rigorous care; the selection of nursing professionals with technical education and hands-on experience can improve the quality of care and patient safety and open new opportunities for participation in patient decision-making.

## Conclusions

This study found that the work engagement status of registered pediatric nurses was relatively high but particularly average in terms of the vigor component. To the best of our knowledge, this research is the first to examine how personal- and work-related variables affect work engagement among pediatric nurses in Madinah, SA. Accordingly, there must be an appropriate number of nurses who are enthusiastic, committed, and engrossed in their work to provide high-quality care. To keep staff turnover to a minimum, nursing directors should pay close attention to the vigor component based on the present findings. It is essential that work features be made more motivating and that practice settings be defined by high levels of support to satisfy the psychological and physical demands of nursing. Specifically, our findings imply that nurse leaders may increase the level of engagement of their staff, especially young nurses, using a wider range of nursing abilities.
